# Assessment of Registration Information on Methodological Design of Acupuncture RCTs: A Review of 453 Registration Records Retrieved from WHO International Clinical Trials Registry Platform

**DOI:** 10.1155/2014/614850

**Published:** 2014-02-12

**Authors:** Jing Gu, Qi Wang, Xiaogang Wang, Hailong Li, Mei Gu, Haixia Ming, Xiaoli Dong, Kehu Yang, Hongyan Wu

**Affiliations:** ^1^Evidence-Based Medicine Center, School of Basic Medical Sciences, Institute of Integrated Traditional Chinese and Western Medicine, Lanzhou University, Donggang West Road, Chengguan, Lanzhou, Gansu 730000, China; ^2^New Product Development of Chinese Medicine Engineering Lab, Gansu University of Traditional Chinese Medicine, Dingxi East Road, Chengguan, Lanzhou, Gansu 730000, China; ^3^Xiaoman Middle School, Zhangye, Gansu 730000, China

## Abstract

*Background*. This review provides the first methodological information assessment of protocol of acupuncture RCTs registered in WHO International Clinical Trials Registry Platform (ICTRP). *Methods*. All records of acupuncture RCTs registered in the ICTRP have been collected. The methodological design assessment involved whether the randomization methods, allocation concealment, and blinding were adequate or not based on the information of registration records (protocols of acupuncture RCTs). *Results*. A total of 453 records, found in 11 registries, were examined. Methodological details were insufficient in registration records; there were 76.4%, 89.0%, and 21.4% records that did not provide information on randomization methods, allocation concealment, and blinding respectively. The proportions of adequate randomization methods, allocation concealment, and blinding were only 107 (23.6%), 48 (10.6%), and 210 (46.4%), respectively. The methodological design improved year by year, especially after 2007. Additionally, methodology of RCTs with ethics approval was clearly superior to those without ethics approval and different among registries. *Conclusions*. The overall methodological design based on registration records of acupuncture RCTs is not very well but improved year by year. The insufficient information on randomization methods, allocation concealment, and blinding maybe due to the relevant description is not taken seriously in acupuncture RCTs' registration.

## 1. Introduction 

As an alternative medicine methodology, acupuncture has been shown to provide definite curative effects [1, 2] and to cause fewer adverse reactions than some other treatment modalities and has been approved by the WHO and by many other medical and health institutions in some western countries [3–6]. It is now practiced widely around the world.

Recently, many researchers have expressed concern regarding the quality of acupuncture clinical trials. These studies revealed that clinical trials of acupuncture had many problems, such as poor methodological quality, unscientific research protocols, and repeated trials of acupuncture [7]. Moreover, their report quality was low [8] and some key information was missing [9]. Problems with publication bias and selective reporting bias had also extended to acupuncture clinical trials. Based on the above findings, the results of acupuncture trails were found to be unconvincing and unreliable [10, 11].

Randomized controlled trials (RCTs) are generally regarded as first-class evidence [12, 13] and the “gold standard” designed to assess the effectiveness of medical intervention. It is due to the application of rigorous methodology, such as using randomization, allocation concealment, and blinding which contribute to eliminating various sources of bias as far as possible and to ensure the inner authenticity of scientific research. However, the poor methodological quality of acupuncture RCTs [14] may make their results unconvincing and unreliable. A possible reason is that the methodological part of RCTs protocol before the experiment started is not perfect enough. To our knowledge, so far there is no study on the assessment of methodological design in protocol of acupuncture RCTs.

Registration can help with transparency of clinical trials by making protocol information and results available to the public. We could acquire the protocols of acupuncture clinical trials by searching in the registration data sets of WHO registries. Examining protocols and examining full reports are two methods for test information-access. Some researchers may tend to get methodology information from full reports [14], but we collected this information from protocols. Besides, full reports are focusing on reporting study results and protocols are mainly about the experimental design. Considering that our study was aimed at investigating the methodology design of RCTs before they were put into practice, examining protocols may obtain information more comprehensive and reliable.

The objective of this study was therefore to assess the methodological design of these protocols (registered records) of acupuncture RCTs. We particularly concerned the information on randomization methods, allocation concealment and blinding.

## 2. Methods

### 2.1. Source of Data

Using the ICTRP Search Portal (http://apps.who.int/trialsearch/Default.aspx), we researched the registration data sets of WHO registries. Registries included the following: Australian New Zealand Clinical Trials Registry (ANZCTR), Chinese Clinical Trial Register (ChiCTR), ClinicalTrials.gov, Clinical Trials Registry-India (CTRI), Cuban Public Registry of Clinical Trials (RPCEC), EU Clinical Trials Register (EU-CTR), German Clinical Trials Register (DRKS), Iranian Registry of Clinical Trials (IRCT), ISRCTN, Japan Primary Registries Network (JPRN), Pan African Clinical Trial Registry (PACTR), Sri Lanka Clinical Trials Registry (SLCTR), the Netherlands National Trial Register (NTR), Clinical Research Information Service (CRiS) public of Korea (KCT), and Brazilian Clinical Trials Registry (ReBec).

### 2.2. Search Strategy

The word “acupuncture” was used as a key word to search in the registration data sets of WHO registries. The search was conducted on data sets from the inception of the registries up to 2 July 2013.

### 2.3. Inclusion Criteria

All records of acupuncture RCTs that were registered in the WHO ICTRP were included. According to the information on the registered titles and study designs, those records which contained “RCT,” “randomized controlled trial,” “randomized,” and “controlled,” were identified as registration records of RCTs and included, while the records contained “single arm study,” “nonrandomized trial,” or other study types (“Cross-sectional study,” “Cohort study,” “Case-control study,” “Crossover design,” etc.) were excluded. For trials with multiple records, data from the record with the earliest registration date was chosen.

### 2.4. Data Extraction

All-inclusive information regarding methodological design was collected from all of the chosen records which showed into the portal and additional information especially on study design and its description was viewed through a hyperlink to the record in the source registry (i.e., the registry that provided the data) published by ICTRP. A small pilot project designed to test the assessment of framework and the training and competence of the data extractors was performed prior to data extraction.

Two researchers (Jing Gu and Xiaogang Wang) extracted the data from each record independently. Disagreements were settled through discussion after data extraction. Data was input into a standardized form that was mainly composed of 3 parts: (1) randomization methods, (2) allocation concealment, and (3) blinding. Conditions that were studied were classified according to the International Classification of Diseases (ICD-10) [15].

### 2.5. Assessment of Information on Methodological Design

Generally, the methodological design for RCT involves whether the randomization methods, allocation concealment, and blinding were adequate or not. In this study, it is performed according to three major procedures in methodological part of the Cochrane Handbook: (1) randomization method: *①* whether the relevant information was mentioned or not and *②* whether randomization method was adequate (referring to a randomized number table, computer software or computerized sequence generation, coin-tossing and dice-rolling, permuted block randomization, etc.) or inadequate; (2) allocation concealment: *①* whether the relevant information was mentioned or not and *②* whether allocation concealment was adequate (central randomization by phone/fax/computer, central allocation, numbered containers, sequentially numbered, opaque, sealed envelopes, etc.) or inadequate; and (3) blinding: *①* whether the relevant information was mentioned or not, *②* whether blinding was adequate (such as single-blind or double-blind was mentioned, and described who was blinded) or inadequate. Methodological quality of acupuncture RCTs was analysed according to the registered year, registries, and the authorization of Ethics Committee (with or without ethics approval).

Descriptive statistics (frequency, percentage) were used to summarize data. Descriptive analysis was performed by Microsoft EXCEL software (version Microsoft Excel 2007; http://office.microsoft.com/zh-cn/). SPSS ware (version 13.0; http://www.spss.com) was used for *χ* ^2^ test.

## 3. Results

### 3.1. Search

**Figure 1:**
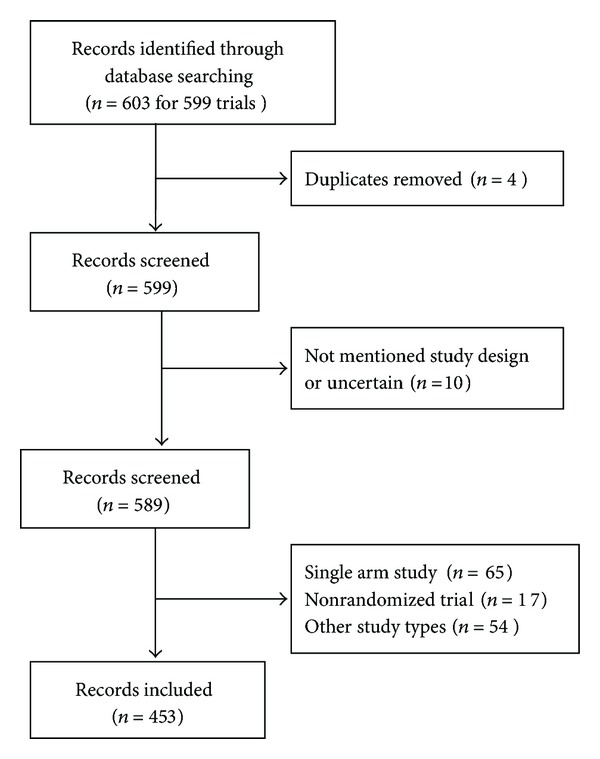
Flow chart of registration records identified, included, and excluded.

A total of 603 records for 599 acupuncture trials were retrieved in the registries. For trials with multiple records, the record with the earliest registration date was used. Four records were excluded. After the primary screening, 146 records were excluded and 453 records were included.  Figure 1 shows the searching steps with respective results. Information on methodological design from the registration of 453 acupuncture RCTs was collected manually from the registered records (Text S1, see the Supplementary Materials available at http://dx.doi.org/10.1155/2014/614850).

### 3.2. General Characteristics

All of the 453 acupuncture RCTs were registered during the period of 1999 to 2013. The number of registrations increased from 1 in 1999 to a peak of 83 in 2012. Registration for 2013 was still in process, so the final number was not yet available. These RCTs were found in the following 11 registries: ClinicalTrials.gov (213), ISRCTN (78), ChiCTR (66), ANZCTR (52), IRCT (16), JPRN (5), KCT (11), EU-CTR (4), DRKS (3), NTR (2), and ReBec (3). No registration records were found in CTRI, RPCEC, PACTR, and SLCTR. Of these included RCTs, all records mentioned health condition(s) or problem(s), of which 11 indicated that the participants were otherwise healthy people or did not specify. The most common conditions were symptoms, signs, and abnormal clinical and laboratory findings (18.5% (84/453)) (Table 1). Synthesizing the information on health condition(s) or problem(s) studied and the primary and key secondary outcomes, we found that 30.9% (140/453) acupuncture trials focused on the treatment of pain.

### 3.3. Overall Assessment of Methodological Design Based on Registration Records of Acupuncture RCTs

Among 453 included registered records of acupuncture RCTs, 107 (23.6%) reported adequate randomization methods, of which 22 mentioned randomized number table, 81 mentioned computer software or computerized sequence generation, and 4 mentioned coin-tossing and dice-rolling; 48 (10.6%) reported adequate allocation concealment, of which 29 mentioned opaque sealed envelopes, 4 mentioned numbered containers, and 15 mentioned central allocation. While other 2 records reported that allocation is not concealed (ACTRN12612000032853, ACTRN12609000698279); 210 (46.4%) reported adequate blinding, of which 136 mentioned single-blind and 147 mentioned double-blind (Table 2).

### 3.4. Changes of Methodological Design with Time

**Figure 2:**
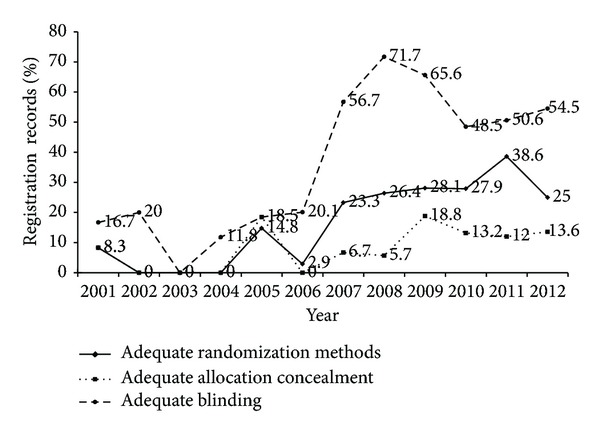
Changes of methodological design of registration with time.

A total of 453 acupuncture RCTs were registered between 1999 and 2013. Overall, the methodological design of these registered RCTs improved year by year; the proportion of adequate randomization methods, allocation concealment, and blinding increased continuously. For adequate randomization methods, the reported proportion increased sharply after 2007 and to the highest of 38.6% in 2011. Regarding adequate allocation concealment, there was a large rise in the proportion after 2007 and to the highest of 18.8% in 2009. In terms of adequate blinding, the reported proportion increased sharply after 2007 and to the highest of 71.7% in 2008 (Figure 2). However, the results of statistical test for trend showed that there were no statistical differences in the presence of adequate randomization methods (*χ* ^2^ = 8.526, *P* = 0.004), adequate allocation concealment (*χ* ^2^ = 8.302, *P* = 0.004), and adequate blinding (*χ* ^2^ = 5.434, *P* = 0.02).

### 3.5. Assessment of Methodological Design of Acupuncture RCTs Registered in Different Registries

**Figure 3:**
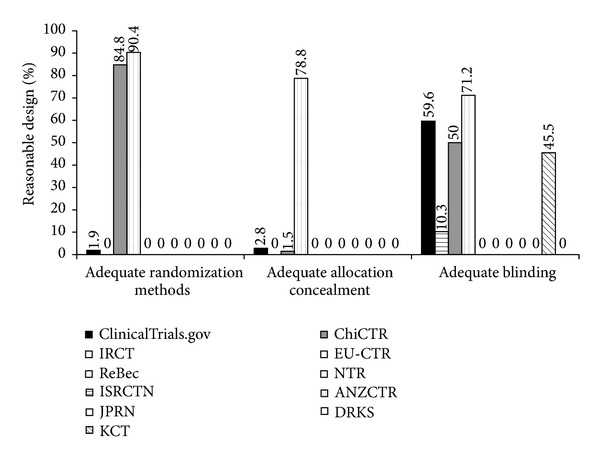
The difference of methodological design of acupuncture RCTs registered in different registries.

Methodological quality was different among registries. Information on the randomization methods and allocation concealment of RCTs was mentioned in registration records from the following 3 registries: ClinicalTrials.gov, ChiCTR, and ANZCTR; the proportion of adequate randomization methods was 1.9%, 84.8%, and 90.4%, respectively, and the proportion of adequate allocation concealment was 2.8%, 1.5%, and 78.8%, respectively. The records from the rest of 8 registries lacked the relate information. Information on blinding was mentioned in the records from the following registries except JPRN: ClinicalTrials.gov, ISRCTN, ChiCTR, ANZCTR, IRCT, KCT, EU-CTR, DRKS, NTR, and ReBec; the proportion of adequate blinding was 59.6%, 10.3%, 50.0%, 71.2%, 0%, 45.5%, 0%, 0%, 0%, and 0%, respectively (Table 2, Figure 3).

### 3.6. The Influence on the Methodological Design of RCTs Registration by the Authorization of Ethics Committee

Information regarding ethics approval was gathered. A total of 126 RCTs have been approved by ethics committees, of which, the proportion of adequate randomization methods, allocation concealment, and blinding were 69.8% (88/126), 31.0% (39/126), and 50.0% (63/126), respectively. While the methodological quality of RCTs without ethics approval (327) was clearly inferior to those with ethics approval, the proportion of adequate randomization methods, allocation concealment, and blinding were only 5.8% (19/327), 2.8% (9/327), and 45.9% (150/327), respectively. According to the statistical results, RCTs without ethics authorization were less likely to provide adequate randomization methods in their registered records than those RCTs with ethics approval (*χ* ^2^ = 206.698, *P* < 0.001). They were also less likely to give out adequate allocation concealment in their registered records (*χ* ^2^ = 77.865, *P* < 0.001). There were no statistical differences in the presence of adequate blinding between RCTs protocol with or without ethics approval (*χ* ^2^ = 0.471, *P* = 0.491) (Table 3).

## 4. Discussion

Clinical trials transparency could contribute to evaluating accurately the authenticity of results and strength of evidence which has ethical and scientific significance. Global clinical trials registration system established and controlled by WHO is a kind of machine-processed transparent which required the implementation and results of trails to be transparent. The methodological design is to guide the whole implementation of the trail and is the fundamental guarantee for the reliability of trail results. So the transparency of methodology is the key to clinical trial transparency which is the necessary part of clinical trials registration.

Unlike general clinical intervention, acupuncture is a special medical technique that treats patients by inserting thin needles into acupoints. Acupuncture clinical practice is mainly concerned with acupuncture theory, acupoints, unique acupuncture manipulation methods, and acupuncture instruments [16]. The “quality” of an intervention in the sense of “how well made is the intervention” is a preclinical not a clinical issue for drugs, biologics, or devices, but for procedures such as acupuncture, intervention quality is a clinical issue. So the reliability of acupuncture effectiveness was more dependent on methodology design of clinical trails when compared with other interventions.

The primary attribution of RCTs is to eliminate selection bias through the method of allocation (which includes randomization and allocation concealment) and to eliminate performance bias or measurement bias through blinding. The quality of a RCT is dependent on the trial design and conduct. The increasing number of researchers realized that RCTs not using randomization, allocation concealment, or blinding exaggerate estimates of effect in different levels. If RCTs not using or inadequate concealment of allocation exaggerate estimates of effect by 30–41% [17, 18]. If the RCTs not using blinding yields 17% larger estimates of treatment effects and in trials with subjective outcomes, effect estimates are exaggerated by 25%. Our study found that the registered acupuncture RCTs were mainly focused on abnormal clinical symptoms or signs, especially on pain management. Their most frequently used measurements of acupuncture effectiveness are subjective indicators, which were influenced largely by not using blinding. A previous study has assessed the methodology quality of published RCTs of traditional Chinese medicine (TCM), 106 (22%) acupuncture RCTs which used adequate randomization methods, 90 (19%) acupuncture RCTs which used adequate allocation concealment, and 198 (41%) which used adequate blinding [14]. Similar results were found in the current study; the methodological design of acupuncture RCTs registered in WHO ICTRP seemed not very well, with insufficient methodological details in their registration records, and the proportion of adequate randomization methods, allocation concealment, and blinding were only 107 (23.6%), 48 (10.6%), and 210 (46.4%), respectively. The lack of methodology information in RCT protocol would increase the subjective casualness of the clinical trial implementation or the influence of artificial factors on the trials. What is more, various possible biase caused by incomplete methodological design of trials would reduce the science and reliability of acupuncture RCTs.

In our research, there were 76.4% registration records that did not provide information on randomization methods, 89.0% lacked information on allocation concealment, and 21.4% were without any information on blinding. So acupuncture RCTs registration records provided less information on methodology, and our study could not draw clear conclusions on “whether acupuncture RCTs methodology was sound or not.” Our results indicated that methodological design failed to catch the attention of acupuncture researchers and registrants neglected the description of randomization methods, allocation concealment, and blinding. There were only 3 register centers in which registration records provided information on randomization methods and allocation concealment, no records from the 8 other register centers had relevant information, which indicates that most register centers do not pay attention to methodological design of RCTs. It is also important to point out that randomization methods, allocation concealment, and blinding were all mentioned in records just from ClinicalTrials.gov, ChiCTR, and ANZCTR. Among them, Clinicaltrials.gov, the center with the largest number of registered acupuncture RCTs, was not the one with the highest rate of sound methodological design and ratio of records of adequate randomization methods and adequate allocation concealment was lower than 5%. While instead we found that ANZCTR had the best quality in the methodology, only 52 acupuncture RCTs were registered in ANZCTR, and most their records provided information on adequate randomization methods, allocation concealment, and blinding. One possible reason was that registration items of ANZCTR which include “methods of sequence generation,” “allocation concealment procedures,” and “blinding” besides 20 items of WHO Trial Registration Data Set (TRDS) [19]. These extra items made detailed requirements for the methodological design of clinical trials, and registration records in ANZCTR may provide sound methodology information to the public. However ClinicalTrials.gov lacks these detailed items of methodology and they are not taken seriously in acupuncture RCTs' registration, so the relevant information provided by records from this center was deficient.

This study indicates that the methodological design of registered acupuncture RCTs improved year by year and it would be specially mentioned that the year of 2007 “looks a turning point;” after that, the proportion of records with adequate randomization methods, allocation concealment, and blinding increased sharply. This increase attributed to using CONSORT (Consolidated Standards for Reporting Trials) [20] and STRICTA (The Standards for Reporting Interventions in Clinical Trials of Acupuncture) [21] to standardize the reporting of clinical trials for many years. CONSORT was established to improve the quality of reporting randomized controlled trials. Acupuncture is a special clinical intervention, CONSORT, as reporting specification fitting in most interventions such as medication and vaccines cannot reflect the characteristic of acupuncture accurately and completely. So STRICTA was proposed on the basis of the CONSORT, which specifically aimed at reporting acupuncture clinical trails. These guidelines are in the form of a checklist for use by authors and journal editors. Checklist emphasizes reporting methodology of RCTs which requires the detailed information on randomization methods, allocation concealment, and blinding and may catch the attention of researchers of acupuncture clinical trials. So, registration information shows that the methodological quality of acupuncture RCTs is yearly improved. Another possible reason why methodological design in registration records after 2007 notably improved was the requirements of clinical trials registration and the opening of a new registry.

We compared the trails with or without ethics authorization, the registration of RCTs approved by ethics committee provided more sufficient methodology information and with relatively well-developed methodological design. Maybe this review mechanism urged trails to accord with ethical requirements in order to ensure its authenticity with scientific and rigorous methodological design.

There were several limitations in the current study. First, “acupuncture” is the only word that was used to search registered acupuncture RCTs; however, other related words like “needling”, “acupressure”, “moxibustion” and “acupoint” are sometimes used so that some acupuncture RCTs related to those words may have been missed. Second, acupuncture RCTs registration records provided insufficient information on methodology, and our study could not draw clear conclusions on “whether acupuncture RCTs methodology was sound or not.”

In conclusion, the overall methodological design based on registration records of acupuncture RCTs is not very well. The insufficient information on randomization methods, allocation concealment, and blinding maybe due to the relevant description is not taken seriously in acupuncture RCTs' registration.

## Supplementary Material

Supplementary Material: Text S1 shows the Main ID of 453 included registration records.

## Figures and Tables

**Table 1:** Types of diseases.

Types of diseases (common ICD-10*)	Number (%) of *n* = 453
Symptoms, signs, and abnormal clinical and laboratory findings, not classified elsewhere	84 (18.5)
Diseases of the musculoskeletal system and connective tissue	65 (14.3)
Mental and behavioural disorders	48 (10.6)
Diseases of the genitourinary system	37 (8.2)
Diseases of the nervous system	38 (8.4)
Diseases of the digestive system	32 (7.1)
Diseases of the circulatory system	30 (6.6)
Neoplasms	22 (4.9)
Diseases of the respiratory system	24 (5.3)
Pregnancy, childbirth, and the puerperium	16 (3.5)
Infectious and parasitic diseases	12 (2.6)
Injury, poisoning, and certain other consequences of external causes	13 (2.9)
Endocrine, nutritional, and metabolic diseases	8 (1.8)
Diseases of the eye and adnexa	8 (1.8)
Diseases of the skin and subcutaneous tissue	3 (0.7)
Diseases of the ear and mastoid process	2 (0.4)
Not specified or healthy people	11 (2.4)

*Common ICD-10: International Classification of Diseases 10.

**Table 2:** Overall assessment of methodological design based on registration records of acupuncture RCTs.

	Overall	ClinicalTrials.gov	ISRCTN	ChiCTR	ANZCTR	IRCT	JPRN	EU-CTR	DRKS	ReBec	KCT	NTR
	453 (%)	213 (%)	78 (%)	66 (%)	52 (%)	16 (%)	5 (%)	4 (%)	3 (%)	3 (%)	11 (%)	2 (%)
Not mentioned randomization methods	346 (76.4)	209 (98.1)	78 (100)	10 (15.2)	5 (9.6)	16 (100)	5 (100)	4 (100)	3 (100)	3 (100)	11 (100)	2 (100)
Adequate randomization methods	**107 (23.6)**	**4 (1.9)**	**0 (0)**	**56 (84.8)**	**47 (90.4)**	**0 (0)**	**0 (0)**	**0 (0)**	**0 (0)**	**0 (0)**	**0 (0)**	**0 (0)**
Randomized number table	22	1	0	16	5	0	0	0	0	0	0	0
Computer software or computerised sequence generation	81	2	0	40	39	0	0	0	0	0	0	0
Coin-tossing and dice-rolling	4	1	0	0	3	0	0	0	0	0	0	0
Not mentioned allocation concealment	403 (89.0)	207 (97.2)	78 (100)	65 (98.5)	9 (17.3)	16 (100)	5 (100)	4 (100)	3 (100)	3 (100)	11 (100)	2 (100)
Adequate allocation concealment	**48 (10.6)**	**6 (2.8)**	**0 (0)**	**1 (1.5)**	41 (78.8)*	**0 (0)**	**0 (0)**	**0 (0)**	**0 (0)**	**0 (0)**	**0 (0)**	**0 (0)**
Opaque sealed envelopes	29	3	0	0	26	0	0	0	0	0	0	0
Numbered containers	4	1	0	0	3	0	0	0	0	0	0	0
Central allocation	15	2	0	1	12	0	0	0	0	0	0	0
Not mentioned blinding	97 (21.4)	12 (5.6)	53 (67.9)	26 (39.4)	1 (1.9)	0 (0)	5 (100)	0 (0)	0 (0)	0 (0)	0 (0)	0 (0)
Mentioned blinding	356 (78.6)	201 (94.4)	25 (32.1)	40 (60.6)	51 (98.1)	16 (100)	0 (0)	4 (100)	3 (100)	3 (100)	11 (100)	2 (100)
Open	70	43	0	6	8	3	0	2	1	0	6	1
Single-blind	136	75	16	22	6	7	0	1	1	3	5	0
Double-blind	147	83	9	12	34	6	0	1	1	0	0	1
Not mentioned who was blinded	73	31	17	1	3	13	0	2	2	3	0	1
Adequate blinding	**210 (46.4)**	**127 (59.6)**	**8 (10.3)**	**33 (50.0)**	**37 (71.2)**	**0 (0)**	**0 (0)**	**0 (0)**	**0 (0)**	**0 (0)**	**5 (45.5)**	**0 (0)**

*Two records reported that allocation is not concealed (ACTRN12612000032853, ACTRN12609000698279).

**Table 3:** The influence on the methodological design of RCTs registration by the authorization of Ethics Committee.

	Without ethics approval (327) (%)	With ethics approval (126) (%)	*χ* ^2^	*P*
Not mentioned randomization methods	308 (94.2)	38 (30.2)		
Adequate randomization methods	**19 (5.8)**	**88 (69.8)**	**206.698**	**0.000**
Not mentioned allocation concealment	318 (97.2)	85 (67.5)		
Adequate allocation concealment	**9 (2.8)**	39 (31.0)*	**77.865**	**0.000**
Not mentioned blinding	72 (22.0)	25 (19.8)		
Mentioned blinding	255 (78.0)	101 (80.2)		
Open	52	18		
Single-blind	103	33		
Double-blind	100	50		
Not mentioned who was blinded	53	20		
Adequate blinding	**150 (45.9)**	**63 (50.0)**	**0.471**	**0.491**

*Two records reported that allocation is not concealed (ACTRN12612000032853, ACTRN12609000698279).
