# Supplementary material for: Assessment of Registration Information on Methodological Design of Acupuncture RCTs: A Review of 453 Registration Records Retrieved from WHO International Clinical Trials Registry Platform
Source: Evid Based Complement Alternat Med. 2014 Feb 12;2014:614850. doi: 10.1155/2014/614850 (PMC3945025; doi:10.1155/2014/614850)
Supplement: Supplementary file 1 — Supplementary Material: Text S1 shows the Main ID of 453 included registration records. [file 614850.f1.pdf]

Text S1 Main ID of all included registration records.

ClinicalTrials.gov (213 records)

NCT01650363	NCT01631500	NCT01369238	NCT01189994	NCT01017211	NCT00142532	NCT00010647	NCT00639977
NCT01643577	NCT01491321	NCT01350570	NCT01178008	NCT01014221	NCT00136669	NCT00065403	NCT00636012
NCT01627444	NCT01487590	NCT00010621	NCT01174394	NCT01013337	NCT00104546	NCT00065234	NCT00635037
NCT01612663	NCT01492738	NCT01331395	NCT01165099	NCT01003470	NCT00094874	NCT00064740	NCT00624793
NCT01614639	NCT01483885	NCT01338701	NCT01162018	NCT01002846	NCT00093327	NCT00060021	NCT00621660
NCT01598974	NCT01481090	NCT01357343	NCT01152632	NCT00245752	NCT00090389	NCT00055354	NCT00610584
NCT01580280	NCT01479920	NCT01315561	NCT01141231	NCT00233857	NCT00090337	NCT00035399	NCT00602940
NCT01573858	NCT01449396	NCT01283477	NCT01124955	NCT00225316	NCT00086021	NCT00034047	NCT00599677
NCT01579786	NCT01442012	NCT01285687	NCT01116453	NCT00186654	NCT00081965	NCT00032422	NCT00608660
NCT01621139	NCT01439412	NCT01275989	NCT01112943	NCT00221247	NCT00071669	NCT00029497	NCT00599586
NCT01570127	NCT01417741	NCT01276028	NCT01105221	NCT00969280	NCT00065975	NCT00011024	

NCT01551654	NCT01411501	NCT01274793	NCT01102816	NCT00969267	NCT00065585	NCT00010478	
NCT01526031	NCT01398930	NCT01275807	NCT01096420	NCT00957112	NCT00860301	NCT00010491	
NCT01512433	NCT01395511	NCT01271595	NCT00320138	NCT00952432	NCT00859365	NCT00010504	
NCT00950482	NCT01389349	NCT01259180	NCT00317291	NCT00945074	NCT00855140	NCT00724763	
NCT00570024	NCT01389622	NCT01247935	NCT00317317	NCT00932061	NCT00843765	NCT00722462	
NCT00598988	NCT01368393	NCT01231776	NCT00312585	NCT00921492	NCT00839592	NCT00010764	
NCT00554879	NCT01360229	NCT01304979	NCT00302185	NCT00917215	NCT00838994	NCT00692328	
NCT00549835	NCT00464425	NCT00404443	NCT01102868	NCT00913354	NCT00797732	NCT00689897	
NCT00544401	NCT00460161	NCT00355329	NCT01079390	NCT00900965	NCT00826345	NCT00685789	
NCT00528125	NCT00459680	NCT00353847	NCT01060553	NCT00892268	NCT00826215	NCT00682162	
NCT00523016	NCT00455182	NCT00352248	NCT01055561	NCT00885586	NCT00826397	NCT00674713	
NCT00508482	NCT00449241	NCT01218243	NCT01054495	NCT00885183	NCT00786214	NCT00655317	
NCT00425412	NCT00432289	NCT01231425	NCT01050075	NCT00882271	NCT00779818	NCT00646633	
NCT00417313	NCT00430378	NCT01205958	NCT01049074	NCT00874133	NCT00813683	NCT00000394	

NCT00414505	NCT00379327	NCT01201642	NCT01047384	NCT00868517	NCT00804089	NCT00002149	
NCT00405158	NCT00375102	NCT00261755	NCT01037894	NCT00200096	NCT00758017	NCT01447329	
NCT00334165	NCT00284492	NCT00260494	NCT01030029	NCT00148577	NCT00746200	NCT00010972	
NCT00328796	NCT00279071	NCT00260637	NCT01022177	NCT00142597	NCT00736411	NCT00010985	

ISRCTN (78 records)

ISRCTN29230777	ISRCTN29932220	ISRCTN98703707	ISRCTN59155637	ISRCTN22366060	ISRCTN18207278
ISRCTN84709751	ISRCTN07165558	ISRCTN02971192	ISRCTN06223266	ISRCTN74414962	ISRCTN48408850
ISRCTN09754699	ISRCTN12585433	ISRCTN84496835	ISRCTN94142364	ISRCTN52683557	ISRCTN84841116
ISRCTN15186354	ISRCTN99496264	ISRCTN13737091	ISRCTN11374571	ISRCTN18249834	ISRCTN22866867
ISRCTN83626912	ISRCTN63787732	ISRCTN07857866	ISRCTN74335441	ISRCTN36780548	ISRCTN92205535
ISRCTN05293321	ISRCTN34405634	ISRCTN49839714	ISRCTN93327878	ISRCTN28687220	ISRCTN27450856
ISRCTN76896018	ISRCTN40932605	ISRCTN59267538	ISRCTN78434638	ISRCTN97373659	ISRCTN16856737
ISRCTN60217348	ISRCTN80312467	ISRCTN49884134	ISRCTN34841555	ISRCTN61381178	ISRCTN01382777
ISRCTN97151578	ISRCTN90807007	ISRCTN40706107	ISRCTN65814467	ISRCTN23245449	ISRCTN61111416

ISRCTN84985339	ISRCTN24863192	ISRCTN41920953	ISRCTN71727409	ISRCTN12159894	ISRCTN96537534
ISRCTN39740785	ISRCTN49335612	ISRCTN59953486	ISRCTN52062666	ISRCTN18747033	ISRCTN80764175
ISRCTN99395260	ISRCTN08827905	ISRCTN32823720	ISRCTN41571810	ISRCTN98448646	ISRCTN88597683
ISRCTN43104115	ISRCTN88008690	ISRCTN25134802	ISRCTN23891318	ISRCTN74259594	ISRCTN74318483

ChiCTR (66 records)

ChiCTR-TRC-12002206	ChiCTR-TRC-11001379	ChiCTR-TRC-10001086	ChiCTR-TRC-09000471	ChiCTR-TRC-08000138
ChiCTR-TRC-12002081	ChiCTR-TRC-11001353	ChiCTR-TRC-10001078	ChiCTR-TRC-09000455	ChiCTR-TRC-08000143
ChiCTR-TRC-12002003	ChiCTR-TRC-11001352	ChiCTR-TRC-10000902	ChiCTR-TRC-08000083	ChiCTR-TRC-08000069
ChiCTR-TRC-12001971	ChiCTR-TRC-11001349	ChiCTR-TRC-10000791	ChiCTR-TRC-08000261	ChiCTR-TRC-07000036
ChiCTR-TRC-12001972	ChiCTR-TRC-11001347	ChiCTR-TRC-10001023	ChiCTR-ORC-08000305	ChiCTR-TRC-07000024
ChiCTR-TRC-11001813	ChiCTR-TRC-11001343	ChiCTR-TRC-10000950	ChiCTR-TRC-08000302	ChiCTR-TRC-07000010
ChiCTR-TRC-11001727	ChiCTR-TRC-11001310	ChiCTR-TRC-09000527	ChiCTR-TRC-08000297	
ChiCTR-TRC-11001719	ChiCTR-TRC-11001256	ChiCTR-TRC-09000519	ChiCTR-TRC-08000276	
ChiCTR-TRC-11001693	ChiCTR-TRC-11001257	ChiCTR-TRC-10000887	ChiCTR-TRC-08000283	

ChiCTR-TRC-11001655	ChiCTR-TRC-11001245	ChiCTR-TRC-10000889	ChiCTR-TRC-08000278	
ChiCTR-TRC-10001609	ChiCTR-TRC-11001201	ChiCTR-TRC-10000792	ChiCTR-TRC-08000225	
ChiCTR-TRC-11001600	ChiCTR-TRC-11001169	ChiCTR-TRC-10000807	ChiCTR-TRC-08000209	
ChiCTR-TRC-11001540	ChiCTR-TRC-10001160	ChiCTR-TRC-10000746	ChiCTR-TRC-08000184	
ChiCTR-TRC-11001554	ChiCTR-TRC-10001146	ChiCTR-TRC-09000507	ChiCTR-TRC-08000176	
ChiCTR-TRC-11001388	ChiCTR-TRC-10001138	ChiCTR-ORC-09000505	ChiCTR-TRC-08000088	

ANZCTR (52 records)

ACTRN12612000719831	ACTRN12611000393954	ACTRN12610000631000	ACTRN12609000985280	ACTRN12608000264381	ACTRN12605000314628
ACTRN12612000705886	ACTRN12611000288921	ACTRN12610000626066	ACTRN12609000928213	ACTRN12608000239369	ACTRN12606000494538
ACTRN12612000706875	ACTRN12611000226909	ACTRN12610000611022	ACTRN12609000923268	ACTRN12608000231347	
ACTRN12612000693820	ACTRN12611000149965	ACTRN12610000340033	ACTRN12609000751279	ACTRN12608000050358	
ACTRN12612000667819	ACTRN12611000113954	ACTRN12610000232033	ACTRN12609000698279	ACTRN12607000499482	

ACTRN12612000607875	ACTRN12611000088943	ACTRN12610000104055	ACTRN12609000676213	ACTRN12607000105448	
ACTRN12612000096853	ACTRN12611000025932	ACTRN12610000043033	ACTRN12609000480280	ACTRN12607000015448	
ACTRN12612000032853	ACTRN12610001052022	ACTRN12609001054202	ACTRN12609000288224	ACTRN12605000766617	
ACTRN12611000761965	ACTRN12610000850077	ACTRN12609001001280	ACTRN12609000095268	ACTRN12605000483651	
ACTRN12611000614998	ACTRN12610000720011	ACTRN12609000989246	ACTRN12609000073202	ACTRN12605000367640	

IRCT (16 records)

IRCT201201108235N1	IRCT201201127117N2	IRCT201107267117N1	IRCT201106036699N1	IRCT201011275181N4	IRCT138811163284N1
IRCT201202088956N1	IRCT201107026934N1	IRCT138706101061N2	IRCT201101133004N4	IRCT138902013767N1	IRCT201112014578N4
IRCT201111218151N1	IRCT201108174242N2	IRCT201104104422N2	IRCT138902222891N1		

JPRN (5 records)

JPRN-UMIN000007773	JPRN-UMIN000006167	JPRN-UMIN000002254	JPRN-UMIN000001277	JPRN-UMIN000000994

KCT (11 records)

KCT0000385	KCT0000269	KCT0000169	KCT0000164	KCT0000130	KCT0000019
KCT0000383	KCT0000195	KCT0000168	KCT0000153	KCT0000071	

EU-CTR (4 records)

EUCTR2008-002343-16-IT	EUCTR2006-004698-86-DE	EUCTR2006-000947-24-GB	EUCTR2006-000810-18-GB

DRKS (3 records)

DRKS00003767	DRKS00003116	DRKS00000164

RBEC 3 (records)

RBR-58yq52	RBR-59f4yr	RBR-5g7xqh

NTR 2 (records)

NTR1987	NTR1613
